# Taxonomic revision of *Sageretia* (Rhamnaceae) from China I: identities of *S. lucida*, S. thea var. cordiformis and *S. yunlongensis*, with the description of a new species *S. ellipsoidea*

**DOI:** 10.3897/phytokeys.179.64750

**Published:** 2021-06-17

**Authors:** Yi Yang, Hua Peng, Hang Sun

**Affiliations:** 1 Laboratory of Subtropical Biodiversity, Jiangxi Agricultural University, Nanchang, Jiangxi 330045, China Jiangxi Agricultural University Nanchang China; 2 CAS Key Laboratory for Plant Diversity and Biogeography of East Asia, Kunming Institute of Botany, Chinese Academy of Sciences, Kunming 650201, China Kunming Institute of Botany, Chinese Academy of Sciences Kunming China

**Keywords:** Granite mountain, limestone mountain, *
Rhamnus
*, woody vine

## Abstract

A taxonomic revision of *Sageretia lucida*, S. thea var. cordiformis and *S. yunlongensis* in China is presented. *Sageretia lucida* is revised in terms of morphological characters (habit, branchlet color, phyllotaxis and rachis length), distribution, habitat, and phenology; S. thea var. cordiformis is raised to *S. cordiformis*; and *S. yunlongensis* is excluded from the genus *Sageretia* and reduced to the synonym of *Rhamnus nigricans*. Furthermore, a new species, *S. ellipsoidea*, is erected based on the paratype collections of *S. lucida.* The new species morphologically differs from *S. lucida* in having reddish brown branchlets, opposite or subopposite phyllotaxis, shorter rachises, and flowering in spring or early summer. *S. ellipsoidea* is factually closest to *S. hamosa* as they share similar woody-vine habit and larger fruit size, and fruiting in winter, whereas the former can be easily recognized based on its smaller leaf blades, fewer lateral veins, shorter rachises, and ellipsoidal or elliptic-ovoid fruits.

## Introduction

*Sageretia* Brongn., the mock buckthorn genus of Rhamnaceae, contains ca. 35 species (Chen and Schirarend 2007). The genus shows a pan-tropical distribution with most members inhabiting subtropical and tropical Asia, and a few in northeastern Africa and tropical America (Mabberley 2008; Yang et al. 2019). Generally, *Sageretia* species are shrubs or woody vines, and usually thrive in disturbed habitats which have poorly developed soils. Many members of the genus have branchlets terminating in woody thorns as a defense against herbivores, and some of them such as *S. gracilis* Drumm. & Sprague and *S. thea* (Osbeck) Johnst. are popular in bonsai gardening. Besides, the drupes of several species are edible, and the leaves are flavonoid-rich and potential substitutes for tea (Chen and Schirarend 2007; Chung et al. 2009; Hyun et al. 2015).

According to Flora of China (FOC; Chen and Schirarend 2007), a total of 19 species and 3 varieties of *Sageretia* are found in the regions south of the Qinling Mountains and the Huai River. Only one species, *S. paucicostata* Maxim., is extensively distributed northward to the Yinshan Mountains. However, two species and one variety are included in a provincial flora but absent in FOC, including *S. cordifolia* Tardieu in Flora Yunnanica (Fan 2006), *S. filiformis* (Roth) G.Don in Flora Xizangica (Chen and Zhou 1986) and Flora Yunnanica, and S. thea var. taiwaniana (Masam.) Y.C.Liu & C.M.Wang in Flora of Taiwan (Liu et al. 1993). Moreover, three species (*S. gongshanensis* G.S.Fan & L.L.Deng, *S. latifolia* Hand.-Mazz. and *S. yunlongensis* G.S.Fan & L.L.Deng) published earlier than FOC (Handel-Mazzetti 1933; Fan and Deng 1995, 1997), have not been included in FOC. Hence, together with the recently published *S. liuzhouensis* Yi Yang & H.Sun (Yang et al. 2017), 25 species and 4 varieties (19 endemic species and 3 varieties) have been recorded in China to date.

We have been studying the taxonomy, molecular phylogeny and biogeography of *Sageretia* since 2014, especially the members in China. A new species, the tropical Asian origin, and three strongly supported clades matching morphological and distributional divergences, had been reported in *Sageretia* in our previous studies (Yang et al. 2017, 2019). In this paper, we present several taxonomic problems in the genus and conduct corresponding revisions. In the process of protologue collation and specimen examination, *S. lucida* Merr., S. thea var. cordiformis Y.L.Chen & P.K.Chou and *S. yunlongensis* were found taxonomically problematic. Specifically, the paratypes of *S. lucida* distinctly differ from the type and actually represent a different species; S. thea var. cordiformis obviously diverges from S. thea var. thea in morphology and molecular phylogeny, and should be raised to a species; *S. yunlongensis* should be categorized in the genus *Rhamnus* L. rather than *Sageretia*. Thus, we here clarify the identities of these and related species.

## Materials and methods

The protologues of all published names and molecularly phylogenetic studies of *Sageretia*, were carefully reviewed and collated. Specimens or digital specimen images from 26 herbaria, including A, CDBI, CSH, CZH, CSFI, E, FJSI, GXMG, HHBG, HITBC, IBK, IBSC, IMDY, K, KUN, LBG, LD, P, PE, SWFC, S, SYS, SZG, UC, US and WU (abbreviations follow Thiers 2020), were examined. In *Sageretia*, the flowers are generally too small (1–2 mm in diam.) and invariable to be used as diagnostic characters, and habit, branch, leaf and rachis characters are primarily applied in classification by contrast (Chen and Schirarend 2007; Yang et al. 2017). Despite morphological characters, habitat and phenology also perform important roles in diagnosis of *Sageretia* species. Thus, the information regarding photos in Plant Photo Back of China (PPBC, http://ppbc.iplant.cn/) and Chinese Field Herbarium (CFH, http://www.cfh.ac.cn/) was also incorporated in the statistics on distribution and phenology of target species. Furthermore, field investigations were conducted at type locations of *S. lucida* and S. thea var. cordiformis to acquire real knowledge about their habit, habitat and phenology.

## Results and discussion

### Taxonomic treatment

#### Rhamnus nigricans

Taxon classificationPlantaeRosalesRhamnaceae

Hand.-Mazz., Anz. Akad. Wiss. Wien. Math.-Naturwiss. Kl. 62: 234–235. 1925.

AA64DA0F-A850-54DD-A62B-6F628537439C

Fig. 1

Sageretia yunlongensis G.S.Fan & L.L.Deng, Sida 16(3): 477, f. 1. 1995. syn. nov. Type: China. Yunnan: Yunlong County, 1300 m, 26 Oct 1987, *Expedition Team 161* (holotype SWFC!)

##### Type material.

**China. Yunnan**: “Beyendjing medium inter Tschuhsiung (Tsuyung) et Yungbei”, 1800 m, 15 May 1915, *Hand.-Mazz. 6311* (holotype WU!; isotypes A [00051422], K [K000729152]).

**Figure 1.**
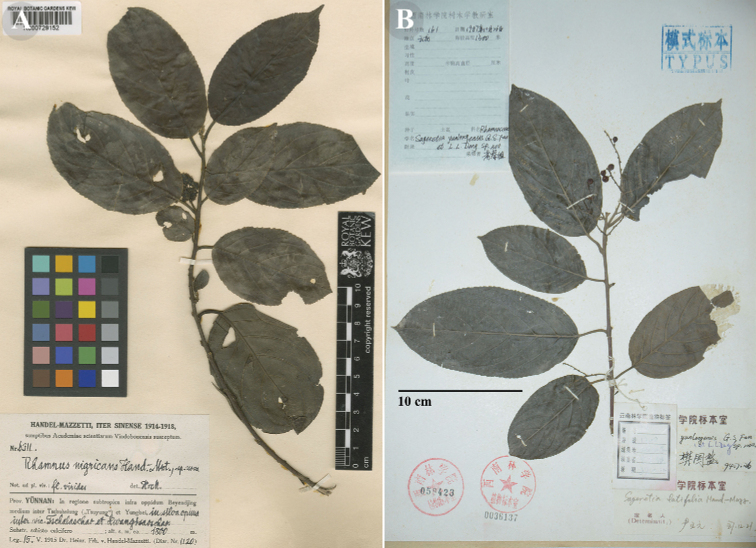
Type specimens of *Rhamnus nigricans* and its synonym **A** isotype of *R. nigricans* (K [K000729152]) **B** holotype of *S. yunlongensis* (SWFC). A obtained from GBIF (https://www.gbif.org/), B photographed by Y. Yang.

##### Description.

Evergreen vines, shrubs or small trees up to 6 m tall, dioecious. Young branches yellowish-brown pubescent; old branches scattered with tuberculate lenticels. Leaves alternate; stipules caducous; petioles 1.2–2.5 cm; leaf blades papery or thickly papery, ovate, oblong to broadly elliptic, 5–16 × 3–7 cm, abaxially puberulent or only on veins, adaxially usually glabrous, lateral veins 5–7 pairs, prominent abaxially, impressed adaxially, base rounded to subcordate, margin densely cartilaginous serrulate, apex acuminate to shortly caudate. Inflorescences axillary, spicate or paniculate, rachises up to 10 cm, puberulent. Flowers unisexual, 5-merous; pedicels 1–2 mm; sepals triangular; petals clawed. Drupes subglobose or globose, ca. 6 mm in diam., turning purple-black at maturity; pyrenes 2–3, asymmetrical, abaxially with a margined furrow extending over 3/4 of length.

##### Phenology.

Flowering from May to July; ripe fruits from October to December.

##### Distribution and habitat.

**Figure 2.**
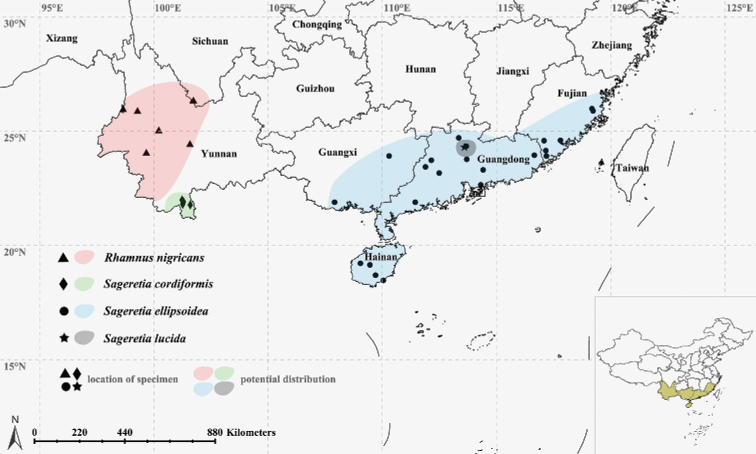
Distribution of *Rhamnus nigricans*, *Sageretia cordiformis*, *S. ellipsoidea*, and *S. lucida.* The sites were obtained from the specimen records, our field investigations and photo information in CFH and PPBC.

The species is distributed in southwestern China (Yunnan; Fig. 2). It grows in thickets on dry slope at elevation from 1300 to 2000 m.

##### Note.

Although the genera *Rhamnus* and *Sageretia* are similar in morphology, they distinctly differ in characters of inflorescence (fascicled, cymose racemes, or cymose panicles in *Rhamnus* vs. spikes or spicate panicles in *Sageretia*) and fruits (basally persistent discoid calyx tube in *Rhamnus* vs. persistent reflexed calyx or remaining inconspicuous disk in *Sageretia*). The type collection of *S. yunlongensis*, *Expedition Team 161*, has branched cymose panicles and fruits basally covered with discoid calyx tube, suggesting it belongs to *Rhamnus* rather than *Sageretia*. In fact, *S. yunlongensis* (Fig. 1B) extremely resembles *R. nigricans* (Fig. 1A), they share similar habit, indumentum, leaf blade shape and size, inflorescence, and fruit, and highly overlapped distribution. Thus *S. yunlongensis* is herein reduced to a synonym of *R. nigricans*.

##### Additional specimens examined.

**China**. **Yunnan**: Weishan Yi and Hui Autonomous County, 1500 m, 2012, *Weishan Expedition Team 5329271259* (IMDY); Yongde County, 1830 m, 9 Jul 2006, *E.D. Liu 170* (KUN); Shuangbai County, 1670 m, 15 Apr 1957, *W.Q. Yin 747* (KUN, LBG, PE); Lushui County, 1544 m, 12 May 2005, *Gaoligong Shan Biodiversity Survey 23964* (E).

#### Sageretia cordiformis

Taxon classificationPlantaeRosalesRhamnaceae

(Y.L.Chen & P.K.Chou) Yi Yang, H.Sun & H.Peng, comb. & stat. nov.

D2EC203A-13F8-53A5-A156-C6648B4487ED

urn:lsid:ipni.org:names:77217740-1

**Figure 3.**
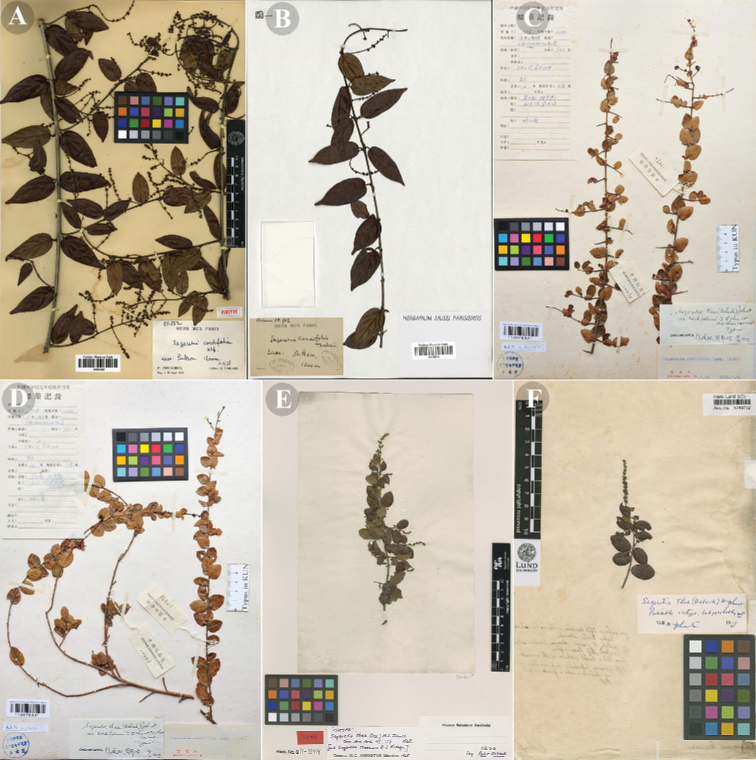
Type specimens of *Sageretia cordifolia*, *S. cordiformis* and *S. thea***A, B** isotypes of *S. cordifolia* (**A**: P [P01818867]; **B**: P [06765093]) **C** holotype of *S. cordiformis* (KUN [1207932]) **D** isotype of *S. cordiformis* (KUN [1207933]) **E, F** isotypes of *S. thea* (E: S [S11-12914]; F: LD [1749752]). **A, B** and **E, F** obtained from GBIF, **C, D** from NOI (https://noi.link/).

**Figure 4.**
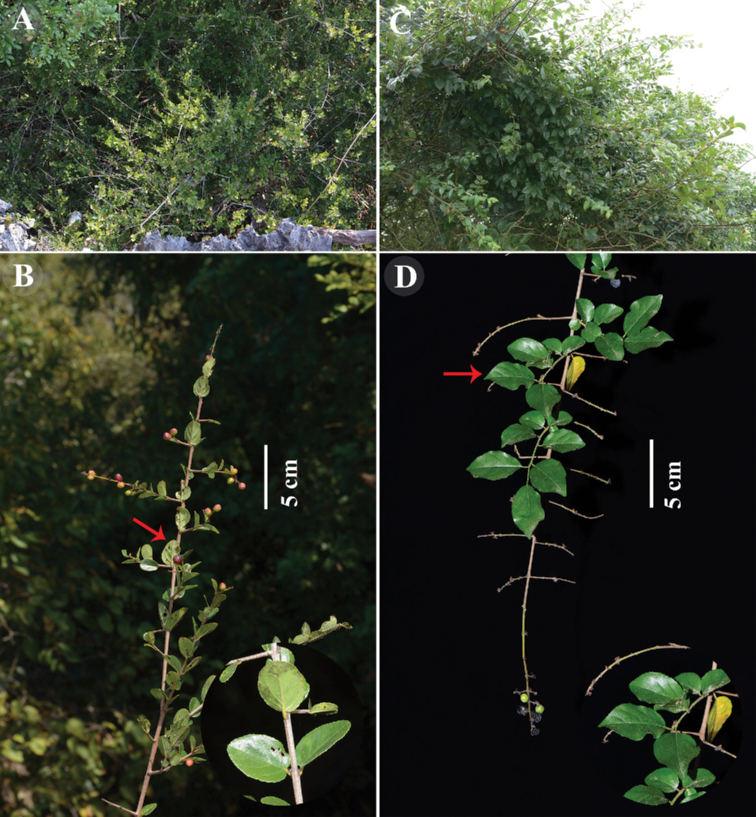
Field photos of *Sageretia cordiformis* (**A, B**) and *S. thea* (**C, D**) **A** and **C** habit **B** and **D** fruiting branch and leaf blades **A–C** photographed by Y. Yang, **D** by X.X. Zhu.

: xin ye que mei teng (心叶雀梅藤) Figs 3C, D , 4A, B

S. thea var. cordiformis Y.L.Chen & P.K.Chou in Bull. Bot. Lab. North-East. Forest. Inst. 5: 74. 1979. Basionym.

##### Type material.

**China. Yunnan**: Xishuangbanna, Mengla County, 730 m, 28 Dec 1958, *W.T. Wang 10496* (holotype KUN [1207932]; isotype KUN [1207933]).

##### Description.

Evergreen shrubs. Branches usually alternate, armed, glabrous to puberulent; second- to fourth-year branches brown. Leaves alternate or subopposite; petioles 1–3 mm, leaf blades leathery, shiny, glabrous, cordate to ovate-orbicular, 1–3 × 0.8–2 cm, lateral veins 2–3 pairs, flat abaxially, impressed adaxially, base cordate or subcordate, margin crenate, apex obtuse or rounded. Inflorescences spicate or spicate-paniculate; rachises 1.5–5 (–10) cm. Flowers sessile; sepals triangular-ovate; petals clawed; stamens as long as petals. Drupes subglobose, ca. 5–6 mm in diam., from green to red and finally turn black, base with persistently reflexed calyx; pyrenes 2–3, emarginate at both ends, asymmetrical.

##### Phenology.

Flowering in September; ripe fruits from December to January of the following year.

##### Distribution and habitat.

The species is distributed in China (southwestern Yunnan; Fig. 2). It grows in thickets on tropical limestone mountains at elevation from 700 to 1100 m.

##### Note.

In Flora Yunnanica, Fan (2006) reduced Sageretia thea var. cordiformis to the synonym of *S. cordifolia* (Fig. 3A, B) with no justification given. *S. cordifolia* occurs in Laos (Pakson) and factually resembles S. thea var thea. However, S. thea var. cordiformis and *S. cordifolia* differ in petiole length (1–3 mm in former vs. 4–8 mm in latter), leaf blade shape (cordate to ovate-orbicular vs. ovate-oblong to ovate-lanceolate) and size (1–3 × 0.8–2 cm vs. 3.5–6 × 1.5–3 cm), and number of lateral veins (2–3 pairs vs. 3–5 pairs) (Table 1). Hence, we disagree with Fan’s treatment.

**Table 1.** Comparison of habitat and morphology among *Sageretia cordifolia*, *S. cordiformis*, and *S. thea* based on field observation, herbarium collections, and photo information obtained from CFH and PPBC.

	*S. cordifolia*	*S. cordiformis*	*S. thea*
**Habitat**	unknown	limestone mountains	hills or mountains
**Branch color**	gray	gray-brown to brownish	brownish
**Petiole length**	4–8 mm	1–3 mm	2–7 mm
**Leaf blade shape**	ovate-oblong to ovate-lanceolate, base cordate, apex acute to caudate-acuminate	cordate or ovate-orbicular, base cordate or subcordate, apex obtuse or rounded	elliptic, oblong, or ovate-elliptic, rarely ovate or nearly orbicular, base rounded or subcordate, apex acute, obtuse, or rounded
**Leaf blade size**	3.5–6 × 1.5–3 cm	1–3 × 0.8–2 cm	2–4.5 × 0.7–2.5 cm
**Leaf texture**	leathery	leathery	paper
**Lateral veins**	3–5 pairs	2–3 pairs	3–5 (–7) pairs
**Rachis**	12–13 cm	1.5–5 (–10) cm	2–12 cm

Based on specimen examination and our field observations, S. thea var. cordiformis and the type variety (Fig. 3E, F, Fig. 4C, D) differ in petiole length (1–3 mm in S. thea var. cordiformis vs. 2–7 mm in type variety), leaf blade texture (leathery vs. papery), and number of lateral veins (2–3 pairs vs. 3–5 (–7) pairs). In fact, Chen and Chou (1979) had clearly mentioned the sharp morphological distinctions between S. thea var. cordiformis and the type variety in the protologue. Furthermore, the results of molecular phylogenetic analyses based on five loci (ITS, ETS, *psb*A-*trn*H, *pet*A-*psb*J and *trn*L-*trn*F) in Yang et al. (2019) indicated the independent species status of S. thea var. cordiformis splitting from the type variety. In their study, S. thea var. cordiformis, *S. pycnophylla* C.K.Schneid. and *S. yilinii* G.S.Fan & S.K.Chen form a clade, while S. thea var. thea is in a highly supported clade with *S. subcaudata* C.K.Schneid. and *S. rugosa*. Considering the broad discrepancies between S. thea var. cordiformis and S. thea var. thea in morphology and molecular phylogeny, we raise the former to *S. cordiformis*.

##### Specimens of *Sageretia cordifolia* examined.

**Laos**. **Pakson**, 1200 m, Nov 1938, *E. Poilane 28562* (holotype P [01818865]; isotypes P [P01818866, P01818867, P06765093]).

##### Additional specimens of *Sageretia cordiformis* examined.

**China**. **Yunnan**: Mengla County, 25 Sept 1961, *Y.H. Li 3588* (KUN); 1200 m, 24 Nov 1975, *Y.H. Li 20033* (HITBC); 1000 m, 9 Sept 1959, *S.C. Pei 10046* (HITBC); 10 Sept 2004, *S.S. Zhou 2084* (PE); ca. 1000 m, 21 Dec 2015, *Y. Yang & Z. Chen xsbn03* (KUN); ca. 1000 m, 24 Dec 2016, *Y. Yang & L.S. Qian OYY001* (KUN).

#### Sageretia ellipsoidea

Taxon classificationPlantaeRosalesRhamnaceae

Yi Yang, H.Sun & H.Peng sp. nov.

7195CBE6-DC65-5CA7-BAAC-121B5D7DDA13

urn:lsid:ipni.org:names:77217741-1

**Figure 5.**
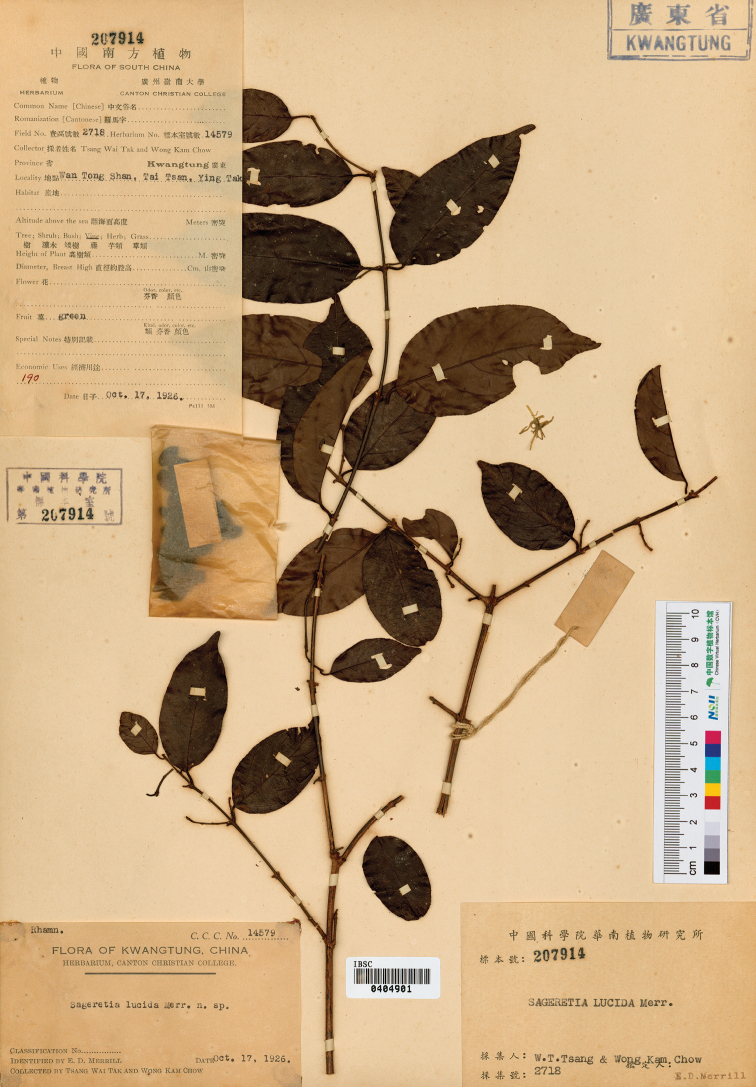
Holotype of *Sageretia ellipsoidea* (IBSC [0404901]). Image obtained from CVH (https://www.cvh.ac.cn/).

**Figure 6.**
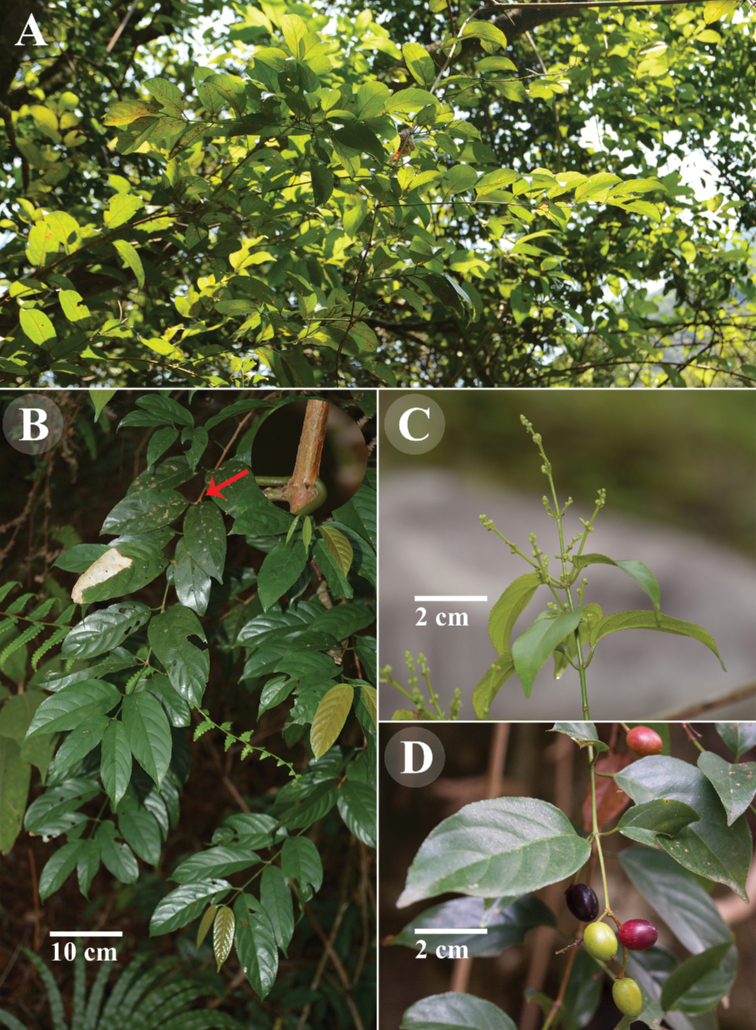
Field photos of *Sageretia ellipsoidea***A** habit **B** branch **C** flowering branch **D** fruiting branch. **A–C** photographed by Y. Yang and **D** by J. Lin.

**Figure 7.**
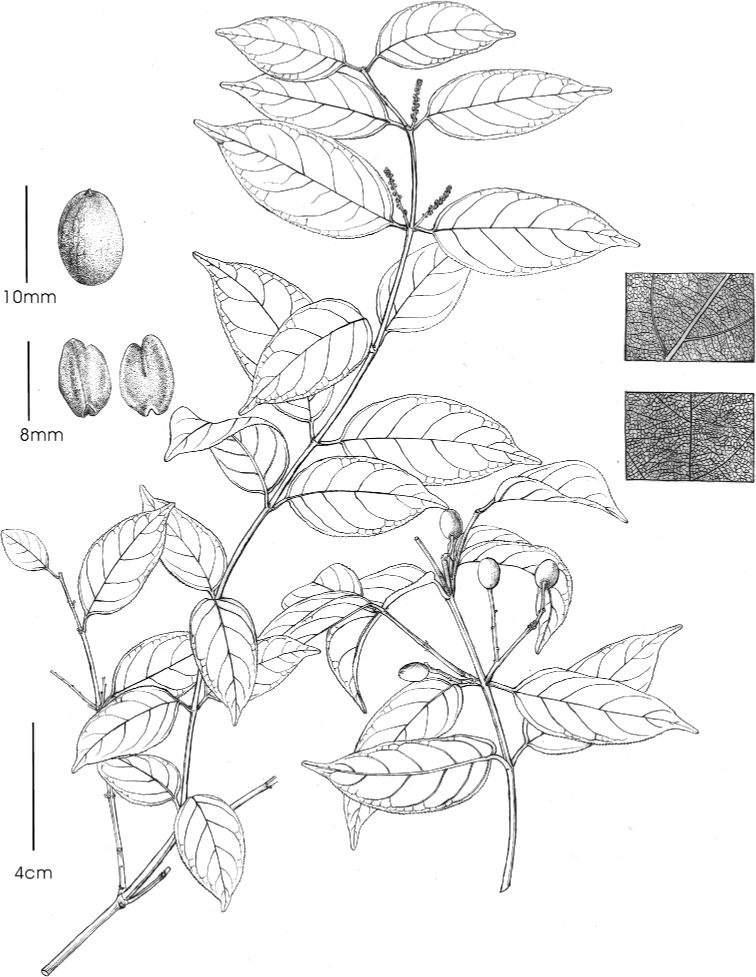
*Sageretia ellipsoidea* Yi Yang, H.Sun & H.Peng **A** flowering branch **B** fruiting branch **C** bottom surface of leaf blade **D** upper surface of leaf blade **E** drupe **F** pyrene (left) and seed (right).

: tuo guo que mei teng (椭果雀梅藤) Figs 5 , 6 , 7

##### Type material.

**China. Guangdong**: “Ying Tak, Taai Tsan, Wan Tong Shan” (Yingde City, Taizhen Town, Wentang Mountain), 17 Oct 1926, *W.T. Tsang & K.C. Wong 2718* (holotype IBSC [0404901]; isotype SYS [SYS00086833]); 18 Oct 1926, *W.T. Tsang & K.C. Wong 2723* (paratype IBSC [0404896]); 6 Oct 1926, *W.T. Tsang & K.C. Wong 2479* (paratype SYS [SYS00086832]).

##### Diagnosis.

Similar to *S. hamosa* (Wall.) Brongn., but differs in having smaller leaves (5–12 × 2.5–4 cm in *S. ellipsoidea* vs. 8–15 (–25) × 3.5–6 (–7) cm in *S. hamosa*), less lateral veins (5–7 pairs vs. 7–11 pairs), shorter rachises (1–3 (–10) cm vs. 6–20 (–25) cm), and ellipsoidal or elliptic-ovoid fruits (vs. subglobose or globose in *S. hamosa*).

##### Description.

Woody vines. Branches opposite or subopposite, glabrous; first-year branches green, sometimes with hard-straight spine opposite to leaf, second- to fourth-year branches reddish brown. Leaves opposite or subopposite; petioles 8–15 mm, leaf blades leathery, ovate-oblong, oblong to elliptic, 5–12 × 2.5–4 cm, lateral veins 5–7 pairs, prominent abaxially, impressed adaxially, base rounded, margin crenate, apex obtuse to shortly acuminate. Inflorescences usually axillary spicate, rarely spicate-paniculate; rachises 1–3 (–10) cm. Flowers subsessile, white to yellowish white; sepals triangular-ovate, ca. 1.5 mm, apex acute; petals clawed; stamens as long as petals. Drupes ellipsoidal or elliptic-ovoid, 10–12 × 5–7 mm, green, turning to orange-red, claret and finally purple-black, base with inconspicuous disk remains; pyrenes 1–2, emarginate at both ends, asymmetrical.

##### Phenology.

Flowering from April to July, ripe fruits from November to January of next-year.

##### Etymology.

This species is named for its ellipsoidal or elliptic-ovoid drupes which are different from other *Sageretia* species (subglobose or globose).

##### Distribution and habitat.

The species is currently found in southern China (Fujian, Guangdong, Guangxi, Hainan; Fig. 2), and probably in northeastern Vietnam. It grows in moist forests along streams on granite mountains below 1200 m.

##### Note.

When he erected the species *Sageretia lucida*, Merrill (1931) cited four collections, including *W.T. Tsang & K.C. Wong 14340*, *14579*, *14584* and *15121*, of which *15121* was selected as type and the other three collections were automatically treated as paratypes. However, the four numbers above belong to herbarium numbers which are ineffective nowadays, and the corresponding field numbers are *W.T. Tsang & K.C. Wong 2479*, *2718*, *2723* and *3260*, respectively. Moreover, another problem is that the paratype collections (*2479*, *2718* and *2723*) factually represent an undescribed *Sageretia* species distinctly differing from *S. lucida* (*3260*) based on geological and morphological evidences. Among the four collections of *S. lucida*, three paratype collections were all collected from “Wan Tong Shan” (Wentang Shan) and type collection from “Chung Tung” (Zhongdong Village, about 10 km apart to the Wentang Shan). Based on field investigations, we find that Wentang Shan has granite landform while Zhongdong Village limestone landform. Furthermore, the three paratype collections are morphologically identical, but noticeably different from the type collection in terms of branchlet color (reddish brown in paratype collections vs. gray to dark gray in type collection), phyllotaxis (opposite or subopposite vs. alternate), rachis length (1–3 (–10) cm vs. 5–10 cm), and phenology (blooming in spring or early summer vs. in autumn) (seen in Table 2). Consequently, the species represented by the paratype collections of *S. lucida* is erected as a new species, namely *S. ellipsoidea* Yi Yang, H.Sun & H.Peng.

Besides, the samples of “*Sageretia lucida*” in Yang et al. (2019) factually also belong to *S. ellipsoidea*. According to Yang et al. (2019), the new species is sister to *S. hamosa* and they form an early diverging clade. In morphology and phenology, the new species also most resembles *S. hamosa* through sharing similar habit (woody vine), larger fruit size (ca. 1 cm long or in diam.) and fruiting season (winter). Nonetheless, *S. ellipsoidea* can be easily distinguished based on its smaller leaves, fewer lateral veins, shorter rachises, and ellipsoidal or elliptic-ovoid drupes (Table 2).

**Table 2.** Comparison of habitat, morphology and phenology (florescence) among *Sageretia ellipsoidea*, *S. hamosa* and *S. lucida* based on field observation, herbarium collections, and photo information obtained from CFH and PPBC.

	*S. ellipsoidea*	*S. hamosa*	*S. lucida*
**Habitat**	granite mountains	non-limestone hills or mountains	limestone mountains
**Habit**	woody vines	wood vines	shrubs
**Phyllotaxis**	opposite or subopposite	alternate or subopposite	alternate
**Branch color**	reddish brown	reddish brown to brown	gray to dark gray
**Petiole length**	8–15 mm	8–15 (–20) mm	6–10 mm
**Leaf blade shape**	ovate-oblong, oblong or elliptic, base rounded, apex obtuse to shortly acuminate	usually oblong or narrowly elliptic, base usually rounded, sometimes cordate, apex caudate-acuminate to shortly acuminate	ovate-oblong to oblong, base subrounded to rounded, apex acuminate
**Leaf blade size**	5–12 × 2.5–4 cm	8–15 (–25) × 3.5–7 cm	5–10 × 2.5–4 cm
**Lateral veins**	5–7 pairs	7–11 pairs	4–6 pairs
**Rachis**	1–3 (–10) cm	6–20 (–25) cm	5–10 cm
**Fruits**	ellipsoidal or elliptic-ovoid	globose or subglobose	unknown
**Florescence**	April to July	July to August	November

##### Additional specimens examined.

**China. Fujian**: Minhou County, 4 Oct 2014, *B. Chen & D.M. Jin CSH12700* (CSH); Nanjing County, 400 m, 19 Nov 1963, *Xiamen Univ. Coll. Team 805* (PE); Pinghe County, 600 m, 23 Feb 1980, *G.S. He 0475* (FJSI); Zhao’an County, 950 m, 16 Mar 2015, *X.F. Zeng ZXF19893* (CZH). **Guangxi**: Jinxiu Yao Autonomous County, 200 m, 6 Apr 1982, *Dayao Shan Expedition Team 13973* (IBSC); Shangsi County, Shiwandashan, 370–390 m, 14 Nov 2011, *D.X. Nong et al. FC2011061* (GXMG). **Guangdong**: Dinghu District, 8 Nov 1963, *G.Q. Ding & G.L. Shi 1132* (IBSC); Fengkai County, 15 Nov 1980, *G.Q. Ding & G.L. Shi 6652* (CDBI); Ruyuan Yao Autonomous County, 17 Aug 1935, *S.K. Lau 23948* (IBK); Huaiji County, 500 m, 26 Oct 1958, *Y.G. Liu 2707* (HHBG); Yangchun City, 6 Nov 1935, *C. Wang 38672* (IBK, PE); Chao’an District, 900 m, 18 Oct 2009, *X.F. Zeng ZXF8404* (CZH); Conghua District, 600 m, 4 Dec 1958, *L. Deng 8836* (IBK); Shenzhen City, 300–350 m, 20 Sept 2006, *G.D. Wang et al. 6474* (SZG); Boluo County, 444.6 m, 1 Apr 2019, *Y. Yang OYY00121* (KUN). **Hainan**: Baoting Li and Miao Autonomous County, 6 May 1935, *F.C. How 72211* (IBK); Changjiang Li Autonomous County, 7 Jun 1934, *H.Y. Liang 64162* (IBK); Baisha Li Autonomous County, 29 Apr 1936, *S.K. Lau 26548* (IBK); Lingshui Li Autonomous County, 21 Oct 1956, *L. Deng 2785* (KUN).

#### Sageretia lucida

Taxon classificationPlantaeRosalesRhamnaceae

Merr. in Lingn. Sci. Journ. 7: 314. 1931.

DFAABBCC-F7B1-5085-9290-9309A1ECDD3B

Fig. 8

##### Type material.

**China. Guangdong**: “Ying Tak, Taai Tsan, Chung Tung” (Yingde City, Taizhen Town, Zhongdong Village), 24 Nov 1926, *W.T. Tsang & K.C. Wong 3260* (holotype UC [319815]; isotypes A [00051501], SYS [SYS00095840], US [00094394]).

**Figure 8.**
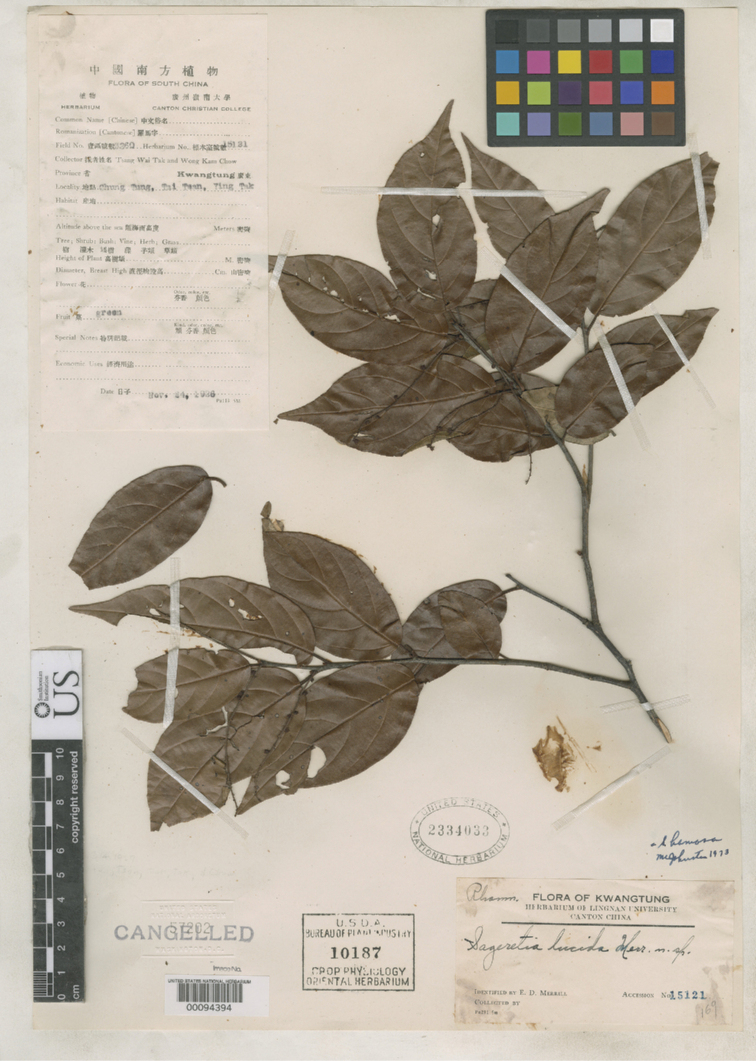
Isotype of *Sageretia lucida* (US [00094394]). Image obtained from GBIF.

##### Description.

Shrubs up to 3 m. Branches alternate, glabrous; second- to fourth-year branches gray to dark gray. Leaves alternate; petioles 6–10 mm, leaf blades leathery, ovate-oblong to oblong, 5–10 × 2.5–4 cm, lateral veins 4–6 pairs, prominent abaxially, impressed adaxially, base sub-rounded to rounded, margin serrulate, apex acuminate. Inflorescences axillary spicate; rachis 5–10 cm. Flowers sessile; sepals triangular-ovate, 1.2–1.5 mm long; petals clawed; stamens as long as petals. Fruits unknown.

##### Phenology.

Flowering in November; fruits unknown, probably ripening from April to May of the next year.

##### Distribution and habitat.

The species is endemic to Yingde, Guangdong, China (Fig. 2). It probably grows in thickets on limestone mountains at elevation ca. 700 m.

##### Note.

*Sageretia lucida* is closest to *S. henryi* Drumm. & Sprague in morphology and sharing similar limestone habitats and flowering in autumn. Thus, Merrill and Chun (1940) synonymized *S. lucida* to *S. henryi*. Chen and Schirarend (2007) disagreed with the synonymization of *S. lucida* because *S. henryi* was factually compared with *S. ellipsoidea* rather than the true *S. lucida* in their study. Nonetheless, we have limited knowledge on *S. lucida* so far because of the lack of field collections, and so know nothing about the fruits. In order to get more information on the species, we conducted investigations at the type location of *S. lucida* (Zhongdong Village) during early summer in 2016 and autumn in 2020, respectively, but failed to find any individuals. Consequently, we suggest to suspend the synonymization of *S. lucida* to *S. henryi* until more evidence has been obtained.

##### Additional specimens examined.

**China. Guangdong**: Yingde City, Zhongdong Village, 13 Nov 1926, *W.T. Tsang & K.C. Wong 3114* (SYS).

## Supplementary Material

XML Treatment for Rhamnus nigricans

XML Treatment for Sageretia cordiformis

XML Treatment for Sageretia ellipsoidea

XML Treatment for Sageretia lucida
